# High HMGB1 levels in sputum are related to pneumococcal bacteraemia but not to disease severity in community-acquired pneumonia

**DOI:** 10.1038/s41598-018-31504-4

**Published:** 2018-09-07

**Authors:** Helena Alpkvist, Simon Athlin, Paula Mölling, Anna Norrby-Teglund, Kristoffer Strålin

**Affiliations:** 1 Department of Infectious Diseases, Karolinska University Hospital, Stockholm, Sweden; 2 Unit of Infectious Diseases, Department of Medicine Huddinge, Karolinska Institutet, Stockholm, Sweden; 3 Department of Infectious Diseases, Örebro University Hospital, Örebro, Sweden; 4 School of Health and Medical Sciences, Örebro University, Örebro, Sweden; 5 Department of Laboratory Medicine, Örebro University Hospital, Örebro, Sweden; 6 Center for Infectious Medicine, Karolinska Institutet, Stockholm, Sweden

## Abstract

During bacterial infections, damage-associated molecular patterns (DAMPs) and pathogen-associated molecular patterns (PAMPs) activate immune cells. Here, we investigated whether plasma and sputum levels of High Mobility Group Box 1 (HMGB1), a prototypic DAMP, are associated with disease severity and aetiology in community-acquired pneumonia (CAP). In addition, in patients with pneumococcal CAP, the impact of the level of sputum *lytA* DNA load, a PAMP, was investigated. We studied patients hospitalised for bacterial CAP (n = 111), and samples were collected at admission. HMGB1 was determined by enzyme-linked immunosorbent assays, and pneumococcal *lytA* DNA load was determined by quantitative polymerase chain reaction. Plasma and sputum HMGB1 levels did not correlate to disease severity (pneumonia severity index or presence of sepsis), but high sputum HMGB1 level was correlated to pneumococcal aetiology (p = 0.002). In pneumococcal pneumonia, high sputum *lytA* DNA load was associated with respiratory failure (low PaO2/FiO2 ratio; p = 0.019), and high sputum HMGB1 level was associated with bacteraemia (p = 0.006). To conclude, high sputum HMGB1 was not associated with severe disease, but with pneumococcal bacteraemia, indicating a potential role for HMGB1 in bacterial dissemination. High sputum *lytA* was associated with severe disease.

## Introduction

High Mobility Group Box 1 (HMGB1) is a nuclear protein that can be actively released by immune cells^1^ or passively released by dead or injured cells^2^. Both infections and injury can cause HMGB1 release^3,4^. Extracellular HMGB1 is a prototypical damage-associated molecular pattern (DAMP). DAMPs are endogenous substances that activate the innate immune cells via pattern recognition receptors (PRRs), resulting in inflammation. However, during bacterial infections, PRRs are at the same time activated by pathogen-associated molecular patterns (PAMPs), i.e., microbial components^3^. It is still unclear how DAMPs and PAMPs co-vary and what their relative contributions are as triggers for inflammation in bacterial infections.

The role of HMGB1 in patients with various bacterial infections is not established. In sepsis, the most severe consequence of bacterial infection, some studies have found a correlation between high levels and disease severity^5^, while others have reported elevated levels without correlation to severity^6,7^. Sepsis can be caused by many different types of infections, of which pneumonia is the most common type^8^. Wang *et al*.^9^ studied plasma HMGB1 levels in 90 patients with suspected community-acquired pneumonia (CAP) and found that it correlated to disease severity, with higher levels in higher pneumonia severity index (PSI) risk classes. Meanwhile, in a study by Angus *et al*.^10^, HMGB1 levels in plasma were similar in CAP patients with or without sepsis. The conflicting results in these studies motivate further studies.

To our knowledge, no previous studies have taken bacterial aetiology into account when studying HMGB1 in CAP. Since bacterial aetiology is important for the clinical course, pathogenesis and severity^11^, we hypothesise that aetiology can be an important factor for HMGB1 levels in CAP.

For patients with severe infection, it has been proposed that HMGB1 release predominantly occurs locally, at the site of infection^12^. Levels of HMGB1 in lower respiratory secretions have been correlated to disease severity in chronic obstructive pulmonary disease (COPD)^13^, asthma^14^, cystic fibrosis^15^, and acute lung injury in *Legionella pneumophila* pneumonia^16^, as well as to low oxygenation index in patients with pneumonia caused by *Pneumocystis jirovecii*^17^. Thus, HMGB1 levels at the site of infection may best reflect the severity of disease in bacterial infections. *Streptococcus pneumoniae* is the most common aetiology of CAP in all severity classes^18,19^. To our knowledge there are no studies on HMGB1 in respiratory secretions from patients with pneumococcal pneumonia.

HMGB1 has been shown to increase the expression of the pro-inflammatory cytokine interleukin 8 (IL-8) in human bronchial epithelial cells^20^. We hypothesise that sputum HMGB1 and plasma HMGB1 levels correlate with plasma IL-8 in patients with bacterial CAP.

To improve our understanding of the role of HMGB1 for inflammation and disease severity in CAP and to compare HMGB1 levels between different aetiologies, we analysed local (sputum) and systemic (plasma) HMGB1 levels in a cohort of well-characterised patients with bacterial CAP. In a sub-analysis of pneumococcal CAP, sputum HMGB1 (a DAMP) was analysed in relation to sputum *lytA* DNA load (a PAMP).

## Material and Methods

### Patients

A total of 235 patients hospitalised for CAP were enrolled in a prospective study^19^ during a 2.5-year period (November 1999–April 2002). The criteria for CAP were acute illness, radiological signs of pulmonary consolidation, and at least two of five of the following signs or symptoms: fever of >38 °C, dyspnoea, cough, pleuritic chest pain, or abnormal lung auscultation. Patients were excluded from the study if they had been hospitalised for any reason during the preceding month.

### Clinical samples and microbiological methods

Samples from blood, sputum, nasopharynx, and urine were collected at admission. Blood cultures were collected in all study patients and were analysed with a Bactec non-radiometric blood culture system (Becton Dickinson, USA). Sputum samples with >5 neutrophils per squamous epithelial cell were considered to be representative of the lower respiratory tract and were subjected to semi-quantitative culturing on blood agar and haematin agar, with incubation in carbon dioxide for 24–48 h^21^. Bacterial pathogens were identified according to standard microbiological methods^22^. The detection limit for a positive sputum culture was 10^5^ colony forming units/mL.

Polymerase chain reaction (PCR) for *Mycoplasma pneumoniae* and *Chlamydophila pneumoniae* were performed on sputum and nasopharyngeal secretions^23^, and detected DNA was considered diagnostic.

The BinaxNOW *Streptococcus pneumoniae* and BinaxNOW *Legionella pneumophila* urinary antigen tests (Alere Inc, USA) were used on non-concentrated urine according to the instructions of the manufacturer, and a positive test was considered diagnostic.

Serology on paired serum samples (acute and convalescent serum, collected at admission and after approximately 4 weeks) was run for detection of *Chlamydophila* species. We used a complement fixation technique, with serology titres of 1:10, 1:20, 1:40, 1:80, 1:160, and 1:320. A >4-fold titre increase between paired sera was considered diagnostic.

### Severity classification

The scoring system of Fine *et al*.^24^ was used for calculating PSI scores. Patients were classified as having severe CAP according to the American Thoracic Society/Infectious Diseases Society of America (ATS/IDSA) criteria^25^, i.e., patients with ≥1 major criteria (invasive mechanical ventilation or septic shock with the need for vasopressors) or ≥3 minor criteria (respiratory rate ≥30 breaths/min, PaO_2_/FiO_2_ratio ≤250, multilobar infiltrates, confusion/disorientation, uraemia (blood urea nitrogen level ≥20 mg/dL), leukopenia (white blood cell count <4000 cells/mm^3^), thrombocytopenia (platelet count <100,000 cells/mm^3^), hypothermia (core temperature, <36 °C), or hypotension requiring aggressive fluid resuscitation). The Sequential Organ Failure Assessment (SOFA) score was calculated at admission. CAP patients with an increase in SOFA score of ≥2 points compared to baseline levels were considered to have sepsis, according to the Sepsis-3 definition^26^.

We prospectively collected data on respiratory rate, oxygen saturation, and administration of oxygen. In the calculation of PaO_2_/FiO_2_ ratio, PaO_2_ was derived from oxygen saturation, according to an oxyhaemoglobin dissociation curve. Data on comorbidities (solid tumour, blood malignancy, liver disease, renal disease, COPD, heart disease, stroke, and diabetes) were collected from the medical records. Mortality data were obtained from the Swedish Population Register.

### Blood samples and sputum samples for HMGB1, IL-8, and *lytA* DNA

Blood samples were collected into endotoxin-free tubes (EndoTube ET*;* Chromogenix AB, Sweden) at admission. The tubes were centrifuged to separate plasma from red blood cells and were then kept at −20 °C until analysis. Sputum samples were mixed with an equal amount of dithiothreitol (Sputolysin®) and frozen at −70 °C.

### HMGB1 analyses

Plasma and sputum samples were analysed with an enzyme linked immunosorbent assay (ELISA) method for HMGB1 (HMGB1 ELISA kit; IBL International), performed in accordance with the manufacturer’s instructions.

### Plasma IL-8

Plasma IL-8 levels were measured with an ELISA (the Human CXCL8/IL-8 Quantikine ELISA kit from R&D Systems), performed in accordance with the manufacturer’s instructions.

### Quantitative *lytA* real-time PCR

The sputum samples were subjected to DNA extraction and quantitative *lytA* PCR, as reported previously^27^. DNA extraction was carried out with the Mag-NAPure Compact Nucleic Acid Isolation Kit I, using a MagNAPure Compact robot (Roche Diagnostics).

The extracted DNA was subjected to quantitative *lytA* PCR, using a LightCycler 2.0 system with Light-Cycler software version 4.1 (Roche Diagnostics). Real-time PCR amplification was performed using the *lytA* forward (CAGCGGTTGAACTGATTGA) and *lytA* reverse (TGGTTGGTTATTCGTGCAA) primers and the P1 (GAAAACGCTTGATACAGGGAGTT-FL) and P2 (LC Red 640-AGCTGGAATTAAAACGCACGAG-PH) probes, with LightCycler FastStart reaction mix hybridisation probes (Roche Diagnostics, Germany). A quantification step was included to calculate the *lytA* DNA load, by using standards with known pneumococcal DNA concentrations.

### Statistical analysis

The statistical analyses were done using SPSS software. The Mann-Whitney U test was used for comparisons between groups in univariate analyses, and the Pearson correlation was used for evaluation of the linear relationship between two continuous variables. Fisher’s exact test was used for comparison of proportions. A p-value below 0.05 was regarded as significant.

### Ethics Statement

The study and all experimental protocols were approved by the Ethics Committee of the Orebro County Council (868–1999). Informed consent was obtained from all participants or their legal guardians. The study was conducted in accordance with the regulations in the Declaration of Helsinki.

## Results

### Study cohort

**Figure 1:**
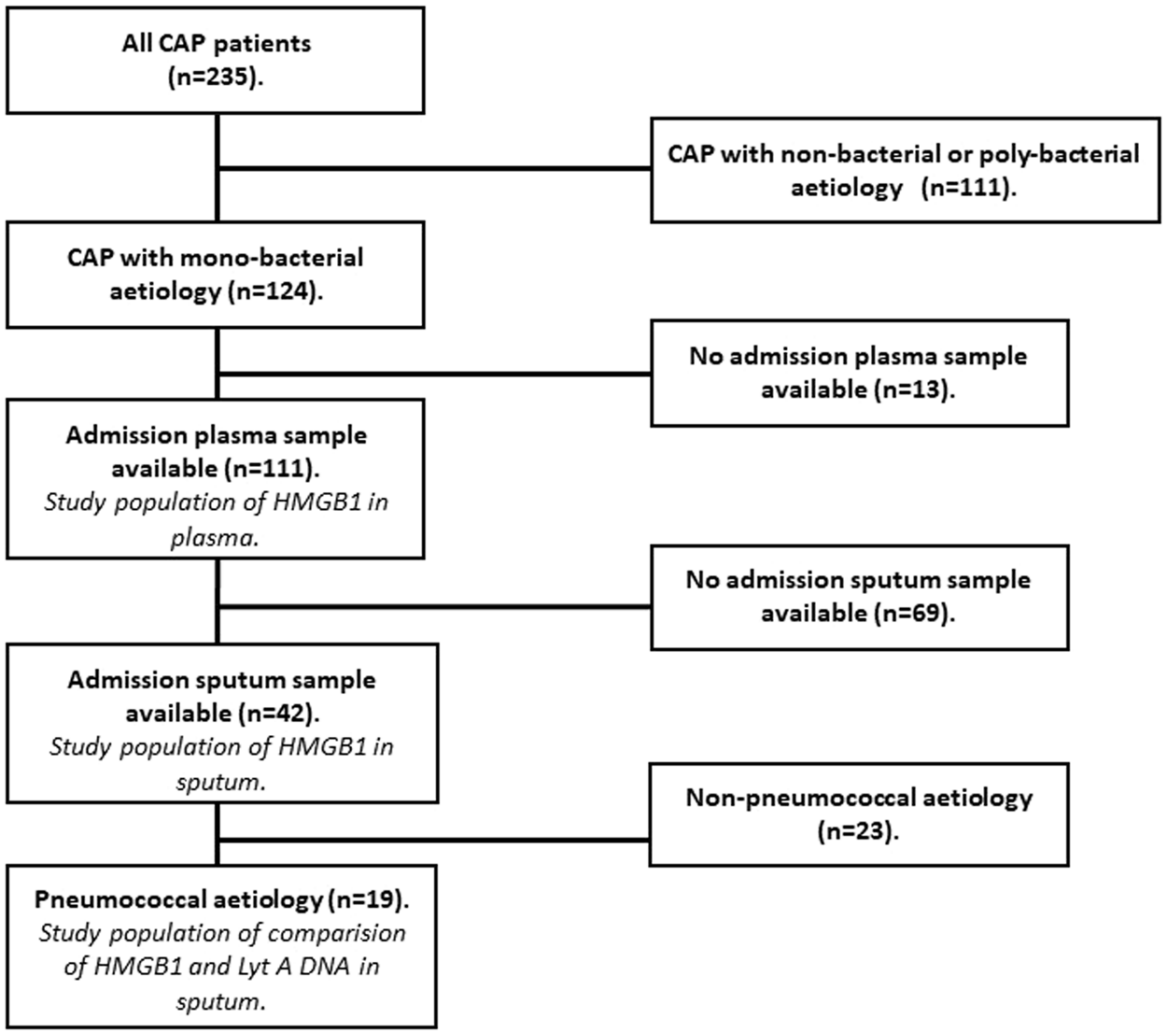
Flow chart of the study population of patients with community-acquired pneumonia (CAP).

Figure 1 presents a flow chart of the study patients. Of the 235 enrolled patients with CAP, 111 patients had mono-bacterial aetiology^19^ and a plasma sample collected at admission (Fig. 1). These 111 patients were included in the study, and an analysis of plasma HMGB1 was performed. There were admission sputum samples available from 42 of the 111 study patients, and those were analysed for sputum HMGB1. Pneumococcal aetiology was diagnosed in 19 of 42 patients with available sputum samples. In all 19 patients, quantitative *lytA* DNA was detected^27^, and they were included in a subgroup analysis for sputum HMGB1 levels and sputum *lytA* DNA load.

Table 1 shows patient characteristics of the study population. Sixty-two (56%) of 111 study subjects were females. The median SOFA score was 2 (range, 0–8), the median PSI score was 78 (range, 18–191), 9 patients (8%) were admitted to the ICU department, and 3 patients (3%) died within 30 days of hospital admission.

**Table 1:** Characteristics of the patient cohort.

Patient characteristics	All patients (n = 111)	All patients with an available sputum sample (n = 42)	All patients with an available sputum sample and pneumococcal aetiology (n = 19)
Female gender, n (%)	62 (56)	22 (52)	7 (37)
Age, median years (range)	67 (18–93)	67 (18–93)	67 (39–89)
Comorbidity^a^, n (%)	50 (45)	22 (52)	12 (63)
Chronic obstructive pulmonary disease, n (%)	19 (17)	12 (29)	8 (42)
Smoking, n (%)	53 (48)	26 (62)	16 (84)
C-reactive protein, median mg/L (range)	214 (9–773)	214 (9–610)	253 (9–610)
Plasma interleukin-8, median pg/mL (range)	32 (0–2009)	33 (2–1516)	46 (2–1516)
PaO_2_/FiO_2_ ratio, median (range)	290 (135–555)	290 (135–458)	300 (135–405)
Severe pneumonia (ATS/IDSA criteria), n (%)	15 (14)	5 (12)	3 (16)
Pneumonia Severity Index risk class IV-V, n (%)	38 (34)	17 (40)	8 (42)
Sepsis (Sepsis-3 criteria), n (%)	67 (60)	29 (69)	13 (68)
Intensive care unit admission, n (%)	9 (8)	4 (10)	2 (11)
30-day mortality, n (%)	3 (3)	1 (2)	0 (0)

^a^Malignancy, liver disease, renal disease, chronic obstructive pulmonary disease, heart disease, history of stroke, diabetes.

Pneumococcal aetiology was detected in 63 patients (57%) by blood culture (n = 22), urinary antigen test (n = 20) or sputum culture (n = 21). *Haemophilus influenzae* aetiology was noted in 16 patients (14%) by blood culture (n = 1) or sputum culture (n = 15). *Staphylococcus aureus* aetiology was noted in 3 patients (3%) all diagnosed by sputum culture. *Mycoplasma pneumoniae* aetiology was diagnosed in 25 patients (23%) by PCR on respiratory secretions. Pneumonia with other atypical bacteria was diagnosed in 4 patients (4%), *Legionella pneumophila* detected in sputum culture (n = 1) or urinary antigen test (n = 1) and *Chlamydophila* species diagnosed with PCR for *Chlamydophila pneumoniae* (n = 1) or serology (n = 1).

### Local and systemic HMGB1 levels in relation to inflammation and disease severity

The median plasma HMGB1 level in all 111 study subjects was 58 ng/mL (range, 6–320 ng/mL). The median plasma HMGB1 level in the 42 CAP patients who had both sputum and plasma samples available was 56 ng/mL (range, 6–320 ng/mL), and the median sputum HMGB1 level was 9 ng/mL (range, 0–563 ng/mL). There was no correlation between HMBG1 levels in sputum and plasma, r = 0.10 and p = 0.55 (Pearson correlation test).

**Figure 2:**
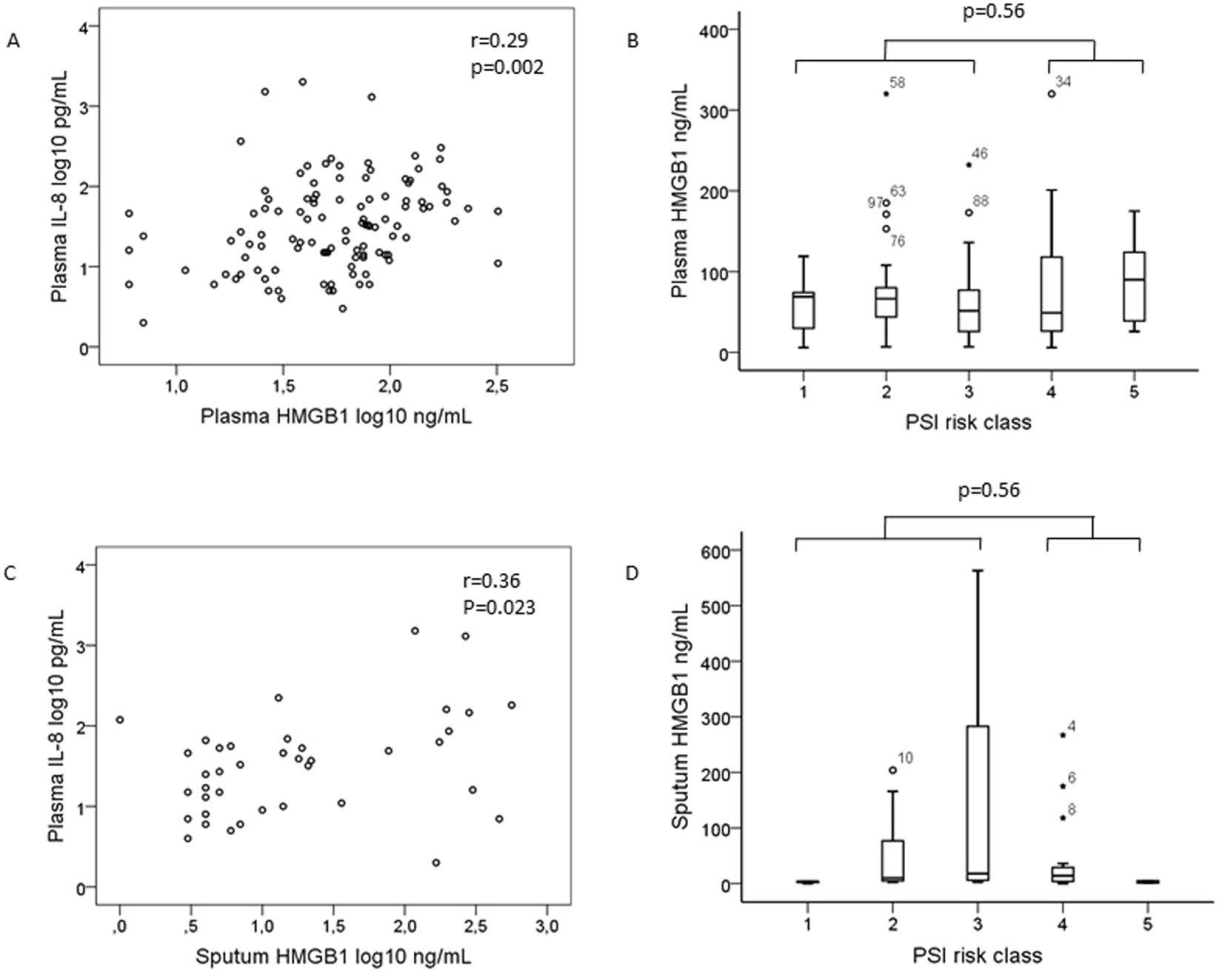
HMGB1 in plasma and sputum in relation to inflammation and disease severity. Correlation between plasma HMGB1 and plasma IL-8 (**A**) or Pneumonia Severity Index (PSI) risk class I (n = 9), II (n = 34), III (n = 30), IV (n = 32) and V (n = 6) (**B**) in 111 patients with bacterial community-acquired pneumonia (CAP). Correlation between sputum HMGB1 and plasma IL-8 (**C**) or PSI risk class I (n = 3), II (n = 9), III (n = 13), IV (n = 15) and V (n = 2) (**D**) in 42 patients with bacterial CAP. In the box plots, the open circles and stars represent outliers and extreme outliers, with patient study number.

There was a weak but significant positive correlation between the levels of plasma HMGB1 and plasma IL-8, r = 0.29, p = 0.002 (Fig. 2A). The median plasma IL-8 concentration was 32 pg/mL (range 0–2009 pg/mL). Plasma HMGB1 levels were not correlated to disease severity as defined by PSI risk class (Fig. 2B), and plasma HMGB1 levels were similar in patients with and without severe pneumonia according to the ATS/IDSA criteria, 44 ng/mL (range 20–173 ng/mL) vs. 59 ng/mL (range 6–320 ng/mL), p = 0.86, and in patients with and without sepsis, according to the Sepsis-3 criteria^26^, 58 ng/mL (range 6–320 ng/mL) vs. 59 ng/mL (range 6–320 ng/mL), p = 0.42. There was no correlation between the plasma HMGB1 level and the PaO_2_/FiO_2_ ratio, r = −0.02 and p = 0.84 (Pearson correlation test).

Similarly, sputum HMGB1 levels showed a weak positive correlation to plasma IL-8, r = 0.36, p = 0.023 (Fig. 2C), but no relation to PSI risk class (Fig. 2D). Additionally, the sputum HMGB1 levels were similar in patients with and without severe pneumonia according to the ATS/IDSA criteria, 5 ng/mL (range 0–300 ng/mL) vs. 10 ng/mL (range 0–563 ng/mL), p = 0.65, and in patients with and without sepsis, according to the Sepsis-3 criteria, 10 ng/mL (range 0–563 ng/mL) vs. 7 ng/mL (range 0–461 ng/mL), p = 0.83. There was no correlation between the sputum HMGB1 level and the PaO_2_/FiO_2_ ratio, r = −0.01 and p = 0.96 (Pearson correlation test).

### HMGB1 levels and aetiology

There was no difference in plasma HMGB1 levels in patients with different bacterial aetiologies (Table 2). However, sputum HMGB1 levels were significantly higher in patient with *S. pneumoniae* aetiology compared to other bacterial aetiologies, median 118 ng/mL vs. 5 ng/mL, p = 0.002 (Table 2).

**Table 2:** Plasma HMGB1 and sputum HMGB1 levels in patients with bacterial community-acquired pneumonia, for different aetiologies.

Aetiology	Plasma HMGB1 median(range), ng/mL	Number of patients	Sputum HMGB1 median (range), ng/mL	Number of patients
*Streptococcus pneumoniae*	51 (6–320)	63	118 (3–563)^a^	19
*Haemophilus influenzae*	53 (20–153)	16	5 (0–15)	13
*Mycoplasma pneumoniae* or *Chlamydophila species*	69 (6–173)	27	8 (0–77)	8
*Staphylococcus aureus*	124 (60–201)	3	12 (1–22)	2
*Legionella pneumophila*	121 (67–175)	2		0

^a^A statistically significant difference between *S. pneumoniae*-infected patients and patients with other aetiologies was shown by the Mann-Whitney U test, p = 0.002.

### HMGB1 levels and patient factors and comorbidities

Univariate analysis of plasma HMGB1 levels and sputum HMGB1 levels, respectively, showed no significant correlations to gender, age ≥65 years old, smoking status, viral co-infection, bilateral infiltrates or pleural fluid.

Patients with COPD tended to have higher sputum HMGB1 levels than patients without COPD, median 17 ng/mL (range 4–563 ng/mL) vs. median 7 ng/mL (range 0–300 ng/mL), p = 0.080, but plasma HMGB1 levels did not differ between the groups (p = 0.33). HMGB1 levels were not correlated to presence of malignancy, heart disease or diabetes. Since only a few patients had liver disease (n = 2), renal disease (n = 2), or history of stroke (n = 3), HMGB1 correlations were not studied for those comorbidities.

### HMGB1 in pneumococcal pneumonia

In 19 patients with pneumococcal CAP, sputum samples were available. Sputum *lytA* DNA was detected in all 19 patients. The median sputum *lytA* DNA load was 7.17 log_10_ copies/mL (range 4.89–8.31 log_10_ copies/mL), and the median sputum HMGB1 concentration was 118 ng/mL (range 3–563 ng/mL). There was no correlation between the sputum HMGB1 level and the sputum *lytA* DNA load, r = −0.37 and p = 0.12 (Pearson correlation test).

**Figure 3:**
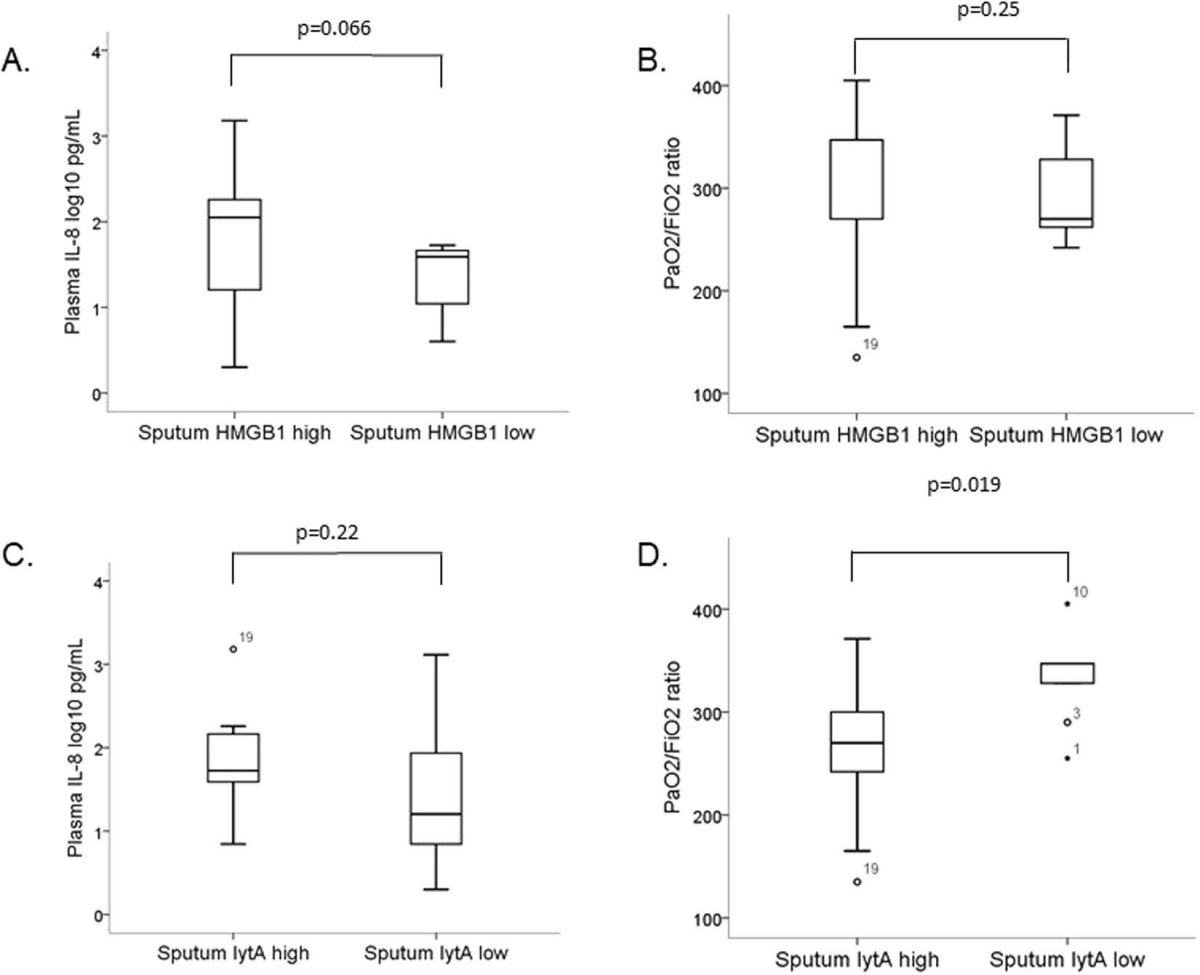
Plasma IL-8 level and PaO_2_/FiO_2_ ratio related to sputum HMGB1 (**A**,**B**) and sputum *lytA* DNA load (**C**,**D**) in 19 patients with pneumococcal community-acquired pneumonia. In the box plots, the open circles and stars represent outliers and extreme outliers, with patient study number.

Patients with sputum HMGB1 levels above or equal to the median tended to have higher plasma IL-8 (p = 0.066), but there was no correlation between the sputum HMGB1 level and the PaO_2_/FiO_2_ ratio (Fig. 3).

Patients with sputum *lytA* DNA loads above or equal to the median had significantly lower PaO_2_/FiO_2_ ratios (p = 0.019), but there was no correlation between the sputum *lytA* load and the plasma IL-8 level (Fig. 3).

**Figure 4:**
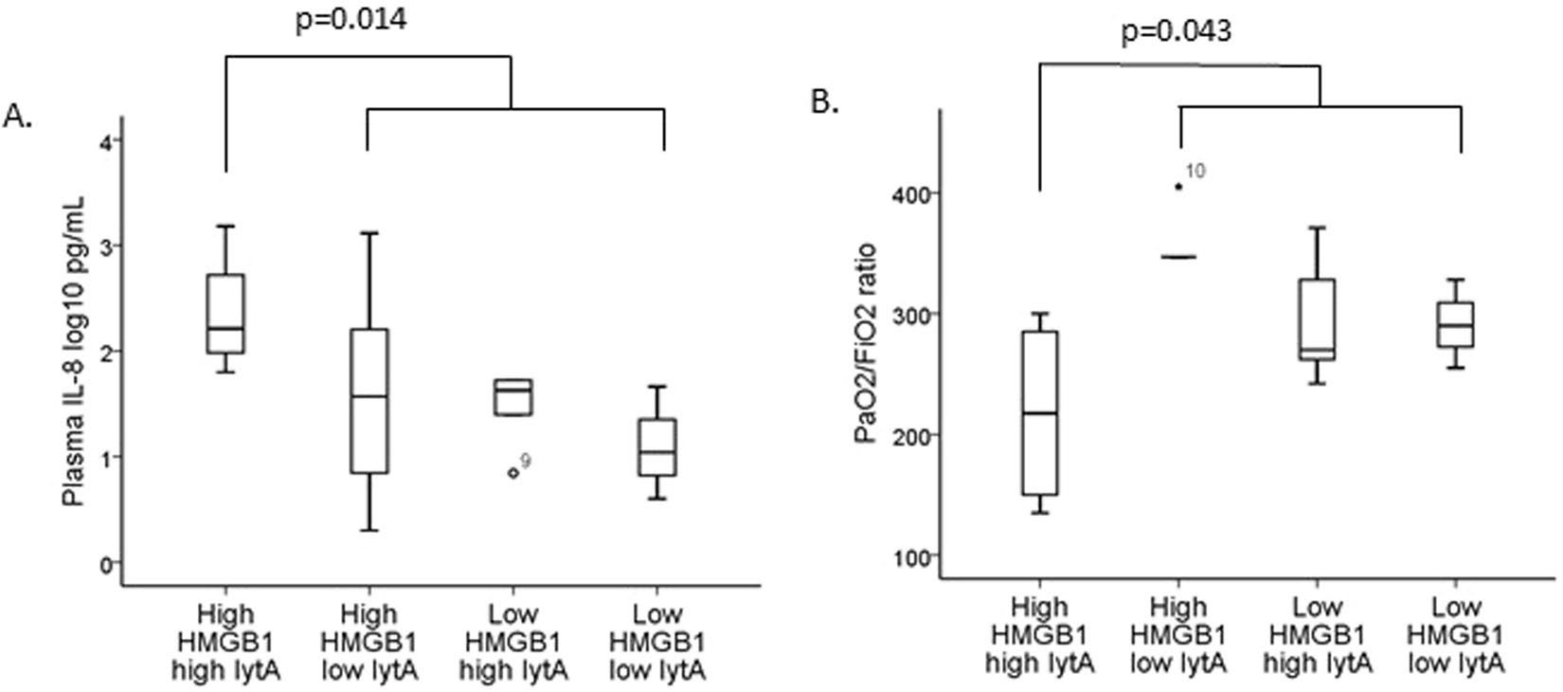
Plasma IL-8 level (**A**) and PaO_2_/FiO_2_ ratio (**B**) in patient groups with different combinations of high and low levels of sputum HMGB1 and sputum *lytA* DNA load in 19 patients with pneumococcal community-acquired pneumonia. In the box plots, the open circles and stars represent outliers and extreme outliers, with patient study number.

When combining the sputum HMGB1 levels and the sputum *lytA* DNA load, we found that patients with high sputum HMGB1 levels (above or equal to the median) and high sputum *lytA* DNA load (above or equal to the median) had significantly higher plasma IL-8 levels (p = 0.014) and significantly lower PaO_2_/FiO_2_ ratios (p = 0.043) compared to the remaining patients (Fig. 4).

**Figure 5:**
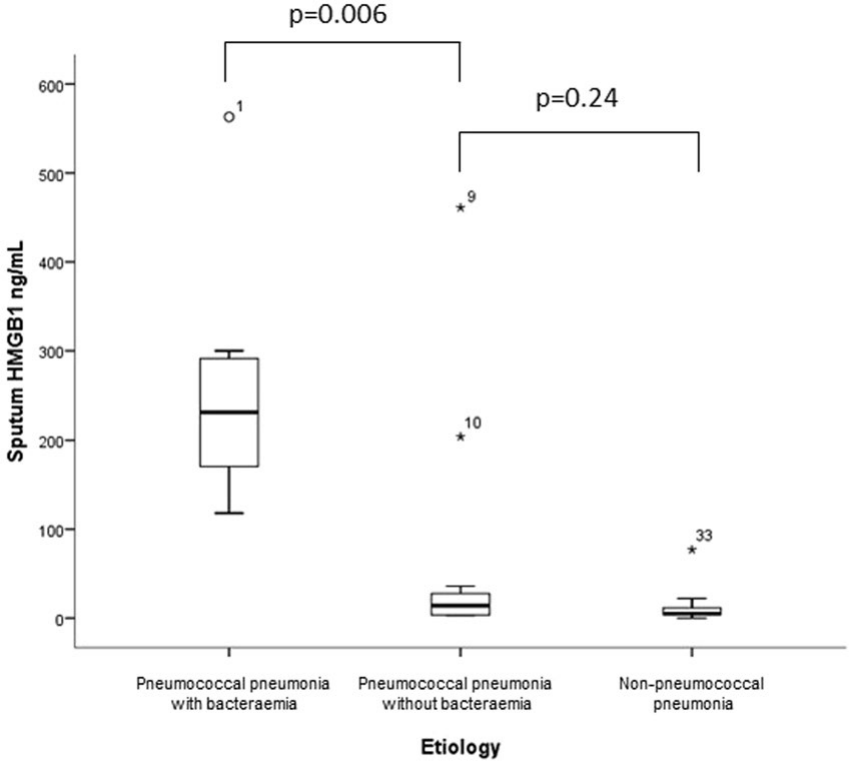
Sputum HMGB1 in patients with bacterial community-acquired pneumonia, comparing the levels of sputum HMGB1 in patients with pneumococcal pneumonia with bacteraemia (n = 8), pneumococcal pneumonia without bacteraemia (n = 11) and non-pneumococcal pneumonia (n = 23). The open circles and stars represent outliers and extreme outliers, with patient study number.

In pneumococcal CAP, patients with pneumococcal bacteraemia had significantly higher sputum HMGB1 levels than patient without pneumococcal bacteraemia, median 232 ng/mL vs. 14 ng/mL, p = 0.005 (Fig. 5). Accordingly, pneumococcal bacteraemia was noted in 8/10 patients with sputum HMGB1 levels above or equal to the median level and in 0/9 of patients with levels lower than the median (p < 0.001). However, there was no difference in median sputum pneumococcal DNA load between patients with and without pneumococcal bacteraemia, 6.74 log_10_ copies/mL vs 7.17 log_10_ copies/mL, p = 0.31.

## Discussion

This study shows for the first time the relationship between plasma and sputum HMGB1 levels and how they vary with aetiology in patients with bacterial CAP. We found that sputum HMGB1 levels were significantly higher in patients with pneumococcal aetiology compared to other aetiologies. We found no correlation between HMGB1 levels in plasma and sputum, and no correlation between HMGB1 and pneumonia severity.

In pneumococcal CAP, patients with both high HMGB1 levels and high *lytA* DNA load in sputum had more severe disease, based on significantly lower PaO_2_/FiO_2_ ratios, than the remaining patients. However, when looking at the biomarkers separately, only the correlation between the PaO_2_/FiO_2_ ratio and the sputum *lytA* DNA load remained significant. Unexpectedly, in patients with pneumococcal CAP, bacteraemia was more frequent in patients with high sputum HMGB1 levels.

Our working hypothesis was that the release of HMGB1 in the lung, secondary to tissue damage, would yield correlating sputum and plasma HMGB1 levels and be associated with disease severity. However, the results did not support this theory. Rather, the findings suggest that the lung is likely not the main source of plasma HMGB1 during bacterial CAP. Our cohort consisted mainly of CAP patients with non-severe pneumonia. On average, we do not expect them to suffer from extensive tissue damage outside the infected lung. Therefore, we hypothesise that immune activation, rather than tissue damage, is the main source of plasma HMGB1 in bacterial CAP.

Our study showed a weak, but statistically significant, correlation between local sputum and systemic plasma HMGB1 levels and systemic levels of the pro-inflammatory cytokine IL-8 in patients with bacterial CAP. This is in agreement with the fact that extracellular HMGB1 activates innate immune cells and is in line with a previous *in vitro* study, where HMGB1 was shown to increase the expression of IL-8 in human bronchial epithelial cells^20^.

We found no correlation between plasma HMGB1 and disease severity in CAP. These results are in agreement with the study by Angus *et al*.^10^, in which similar HMGB1 levels in uncomplicated pneumonia and pneumonia with sepsis were found, but conflict with the result of Wang *et al*.^9^, who found HMGB1 to be correlated to PSI risk class. It is difficult to compare our study with the Wang study, as their study lacked information about X-ray findings, comorbidity, and aetiological information.

To our knowledge, there are no previous studies on HMGB1 in sputum from patients with pneumonia. As HMGB1 levels in sputum of patients with COPD^13^, asthma^14^, and cystic fibrosis^15^, and HMGB1 levels in bronchoalveolar lavage of patients with *L. pneumophila* pneumonia^16^, have been linked to disease severity^28^, we hypothesised that the level of HMGB1 in sputum would be related to pneumonia severity. However, we found no correlation between HMGB1 levels and severe disease, defined by the PSI score, ATS/IDSA criteria, sepsis (defined by Sepsis-3) or PaO_2_/FiO_2_ ratio. In our study, only two patients had *L. pneumophila* pneumonia and neither of them had a sputum sample available for this study, so we cannot compare our results with the study of Higa *et al*.^16^.

High sputum HMGB1 levels were associated with pneumococcal aetiology. However, when comparing patients with pneumococcal CAP with and without bacteraemia, we found that high levels of sputum HMGB1 were primarily seen in patients with pneumococcal bacteraemia. There was no difference in sputum HMGB1 levels between patients with pneumococcal CAP without bacteraemia and patients with non-pneumococcal CAP. Furthermore, there was no correlation between sputum pneumococcal DNA load and bacteraemia. We found this most interesting since our hypothesis was that a high bacterial load in sputum would increase the risk for bacteraemia. There are several plausible explanations for the correlation between high sputum HMGB1 levels and pneumococcal bacteraemia. First, HMGB1 has been shown to increase endothelial permeability^29^. Second, high concentrations of DAMPs have been linked to reduced immune function^4^. For instance, in mice with *Pseudomonas* infection and hypoxic lung damage, treatment with a neutralising anti-HMGB1 monoclonal antibody resulted in an increase in leukocyte phagocytic activity compared with mice receiving control mAb, and this improved phagocytic function was associated with reduced concentrations of airway HMGB1^30^. The correlation between phagocytic activity and concentrations of extracellular HMGB1 was also observed in cultured macrophages. Thus, in a pneumonia lung, high levels of airway HMGB1 may impair bacterial killing. Third, HMGB1 is a marker for tissue damage, and high sputum HMGB1 levels could be secondary to cell and tissue injury in the lungs resulting in impaired mucosal barrier function and thereby facilitating the spread of bacteria from the lungs into the bloodstream.

In the present study, HMGB1 levels were not correlated to the presence of comorbidities, apart from COPD. Patients with COPD tended to have higher levels of sputum HMGB1 than patients without COPD. Accordingly, in induced sputum samples of outpatients, Hou *et al*.^13^ found that high sputum HMGB1 levels were significantly correlated to the presence of COPD^13^.

Both DAMPs and PAMPs activate PRRs to start inflammatory processes. To our knowledge, it has not been clarified which pattern, DAMP or PAMP, is most important for this process in pneumococcal pneumonia^31^. The present study showed that the combination of a high sputum HMGB1 level and a high sputum *lytA* DNA load was associated with inflammation (IL-8 elevation) and respiratory failure (reduced PaO_2_/FiO_2_ ratio) in patients with pneumococcal CAP (Fig. 4). However, when doing separate analyses of DAMP and PAMP, we found that respiratory failure was driven by the *lytA* DNA load (Fig. 3), and the PaO_2_/FiO_2_ ratio was not at all related to the HMGB1 level in univariate analysis (Fig. 3). These findings are in line with our previous study^32^, in which a high pneumococcal DNA load in the respiratory tract was correlated to disease severity in pneumococcal CAP.

This study has limitations. Most importantly, the patient cohort is small, and it is a single cohort. Further studies are needed to examine how the combination of PAMPs and DAMPs, locally at the site of infection as well as in circulation, influence disease severity, the inflammatory response and the immunological response in CAP. One major limitation is that the samples were frozen for several years before analysis. We cannot rule out that the sample conditions have affected the median HMGB1 levels. However, the samples were prospectively collected and stored in a structured and systematic way and they were frozen for a similar period of time. Thus, if the freezing time would have affected the samples, we believe that it would have affected the samples in a similar way and that it would not cause a substantial variation within the cohort. It should be noted that there is a considerable variation in plasma HMGB1 levels between different studies. The study by Angus *et al*.^10^ shows similar plasma HMGB1 levels to our study, whereas the study by Wang *et al*.^9^ shows lower levels. Standardisation of the methods of measuring circulating levels and airway levels of HMGB1 is needed for further research and/or clinical use.

## Conclusions

The present study found that high sputum *lytA* load, but not high sputum HMGB1 level, was associated with more severe disease. However, high sputum HMGB1 level was associated with bacteraemia in pneumococcal pneumonia, indicating a potential role for HMGB1 in bacterial dissemination.

## Data Availability

The datasets generated and analysed during the current study are available from the corresponding author upon reasonable request.
